# How is Telemedicine perceived? A qualitative study of perspectives from the UK and India

**DOI:** 10.1186/1744-8603-7-17

**Published:** 2011-05-20

**Authors:** Melisa Martínez Álvarez, Rupa Chanda, Richard D Smith

**Affiliations:** 1 Department of Global Health and Development, London School of Hygiene and Tropical Medicine; 2 Indian Institute of Management Bangalore, Bannerghatta Road, Bangalore, India

## Abstract

**Background:**

Improvements in communication and information technologies have allowed for the globalisation of health services, especially the provision of health services from other countries, such as the use of telemedicine. This has led countries to evaluate their position on whether and to what extent they should open their health systems to trade. This often takes place from the context of multi-lateral trade agreements (under the auspices of the World Trade Organisation), which is misplaced as a significant amount of trade takes place regionally or bi-laterally. We report here the results of a qualitative study assessing stakeholders' views on the potential for a bi-lateral trade relationship between India and the UK, where India acts as an exporter and the UK as an importer of telemedicine services.

**Methods:**

19 semi-structured interviews were carried out with stakeholders from India and the UK. The themes discussed include prospects on the viability of a bi-lateral relationship between the UK and India on telemedicine, current activities and operations, barriers, benefits and risks.

**Results:**

The participants in general believed there were good prospects for telemedicine trade, and that this could bring benefits to "importing" countries in terms of cost-savings and faster delivery of care and to "exporting" countries in the form of foreign exchange and quality improvement. However, there were some concerns regarding quality of care, regulation, accreditation and data security.

**Conclusions:**

There is potential for trade in this type of health services to succeed and bring about important benefits to the countries involved. However, issues around data security and accreditation need to be taken into consideration. Countries may wish to consider entering bi-lateral agreements, as they provide more potential to address the concerns and capitalise on the benefits. Finally, this paper concludes that more data should be collected, both on the volume of telemedicine trade and on the impact it is having on health systems, as currently there is very limited data on this.

## Background

With increasing globalisation countries have been opening their service sectors to international trade. Health services are no exception to this. Although most trade in health services occurs through regional and bi-lateral routes [1], research on this topic has centred on multi-lateral agreements signed under the World Trade Organisation's General Agreement on Trade in Services (GATS) [2]. This multi-lateral agreement is a system of rules through which international trade in services occurs, and by which all member countries trade with each other. There are fears that trade in health services will accelerate privatisation of health systems [3] and hinder domestic health policy decision-making [4]. On the other hand, some studies have highlighted that increased health services trade and outsourcing can facilitate the sharing of ideas and reform, and improve the sustainability of health systems [5]. However, there is little data to support or refute these claims.

This study is set in the context of a specific mode of trade in health services, *telemedicine*. Telemedicine is the application of telecommunications technology to deliver health services at a distance [6]. Telemedicine trade is already taking place on a large scale, with the global market estimated to be worth $5.8 billion in 2007, and projected to grow to $13.9 billion by 2012 [7]. There are different types of health care services that can be outsourced, although the most important ones are tele-radiology and tele-pathology. These involve scans or samples being sent to radiologists or pathologists for interpretation in a remote location, who then send the results back to the physician. A sector of telemedicine that has received increasing attention is tele-care. Tele-care involves the use of information and communication technologies to monitor patients (often suffering from chronic diseases) from their home. It has already been trialled in United States [8], Chile [9] and Taiwan [10]. Further, there are plans to establish tele-care initiatives in remote parts of Scotland and Canada.

The growing use of telemedicine has prompted a body of literature on the benefits and concerns associated with it. The authors have previously conducted a literature review on telemedicine, exploring the issues that arise from cross-border trade globally, and specifically using the UK and India as a case study [11]. This review found that while trade in telemedicine services has the potential to benefit both the exporting and importing countries by allowing for faster service delivery and raising foreign exchange, there are concerns regarding data security, recognition of qualifications and legal liability [12]. The literature review had two main conclusions. First, very little data was found on the amount of trade in telemedicine that is currently taking place or on the issues associated with it. It is therefore important that more studies are carried out on this, both at the global and at the country level. Second, that were countries to consider this type of trade in health services from a bi-lateral perspective, they could maximise the benefits and minimise the concerns. This is because a contract would be drawn out between the two countries, where conditions can be agreed upon, such as outlining data regulations and security, litigation procedures and liability, and regulation and recognition of qualifications.

With the aim of contributing to the limited evidence base on cross-border trade of telemedicine services, the authors conducted semi-structured interviews with stakeholders in both India and the UK, to seek their opinions on the prospects for a telemedicine trade relationship between these two countries, where India would provide telemedicine services to the UK. Further, the potential benefits and barriers that may arise from this type of relationship were assessed. In doing this, the authors have undertaken a social science perspective to address a gap in the knowledge available regarding telemedicine trade, and hope to contribute to the limited data available to inform countries considering opening their health services to international trade. This paper describes the methodology used to carry out the research, followed by the results from the interviews and the discussion. The paper concludes with key messages.

## Methods

A total of 19 interviews were carried out with stakeholders; of these, eight were from the UK and 11 from India (see table 1). The stakeholders that took part in the study were identified as important from the literature review carried out previously [11]. A snowball sampling technique was then used to identify the other stakeholders. The interviews were semi-structured [13] to allow for additional issues not identified in the literature review to be included in the study. As such, a qualitative instrument was designed based on the findings from the literature review (see Additional File 1 for an example). The instrument was adapted for each stakeholder as appropriate.

**Table 1:** List of stakeholders that took part in the semi-structured interviews.

Stakeholder group	Country	Stakeholder Number
Department of Health	UK	1, 2, 3, 4

Healthcare provider	UK	5, 7

Healthcare provider	India	9, 11, 12, 14, 15, 17, 18

Think tank	UK	8

NGO	India	10

Academic	India	16

Industry association	UK	6

Industry association	India	13

The interviews were carried out by one of the authors in India (RC) and another author in the UK (MMA). The interviews were mainly conducted face-to-face, although some of the Indian participants were interviewed over the phone. Answers were recorded by hand and typed up. The interviewers were in regular contact during the interview process to discuss emerging themes. The interviews were examined using content analysis [14]. Briefly, each script was reviewed and coding categories were identified. The scripts were then analysed using these coding categories. The coding framework consisted of both emergent and a priori coding categories [14]. Coding categories were designed based on the themes identified during the literature review [11]. The transcripts were then reviewed and any new coding categories that arose was incorporated. Any new themes that emerged were explored across all the interview transcripts. This process was iterated until no more new categories emerged (see table 2 for a full list of all coding categories). The coding framework was developed by MMA and reviewed by the other authors until an agreement was reached. Once the list of coding categories was compiled, the interview transcripts were analysed using these codes. The coding units were any sentences or paragraphs that fell under any of the coding categories. The analysis was conducted by MMA, although any ambiguities identified were discussed with the other authors.

**Table 2:** Coding categories and frequency by group

**Coding Category**^**1**^	Frequency by groups							
	**Government officials**		**Healthcare providers**		**Industry associations**		**Others **^**2**^	

	**UK**	**India**	**UK**	**India**	**UK**	**India**	**UK**	**India**

Prospects	2	1	1	5	1		1	1

Sectors	2			8	1	1	1	1

Advantages			1	1	1			2

Regulation	1			4		1		

Quality	2		1	1	1			

Litigation	2			2				

Data safety	1		1	4				

Other barriers	1		1	5	1		1	1

Policy issues	1			5	1			1

^1 ^The coding categories were identified from the interview transcripts. They are described here. **Prospects**: the participants were asked their opinion on telemedicine trade globally and between the UK and India. **Sectors**: the stakeholders were asked to identify which sectors within telemedicine would be most successful from an international trade perspective. **Advantages**: Participants were asked what advantages trade in telemedicine would bring to the countries involved. **Regulation**: the respondents identified regulation as a key barrier to trade in telemedicine. **Quality**: similarly, stakeholders were concerned about the standards of care that telemedicine could offer, especially if provided from another country. **Litigation**: many participants were concerned about the legal implications of malpractice in cross-border telemedicine. **Data safety**: restrictions from the European Union (EU) on India's data management were highlighted as a key constraint. **Other barriers**: this category summarises all the other barriers identified by the stakeholders interviewed. **Policy issues**: respondents were asked to identify policy changes that would be needed for a relationship between the UK and India to take off.

^2 ^Think tanks, NGOs and academic institutions

## Results

A summary of all the coding categories and their frequency according to stakeholder is shown on table 2.

### Current activities

The participants were asked about the current cross-border telemedicine activities their country engages in. The respondents highlighted that both the UK and India engage in international trade of telemedicine. The UK was said to have arrangements for the remote provision of diagnostic services with South Africa and Belgium, whereas India was reported to outsource health services to Singapore.

However, there was disagreement with regards to current activities between the UK and India amongst the UK stakeholders, as some believed the UK does not import any telemedicine services from India while others believed that it does.

*There have already been agreements to outsource back office work, and some contracts have been with India. These contracts have been running for two years ... But so far, it has only been the non-clinical work that has been outsourced *(Respondent #4)

*The UK is currently outsourcing its back office work to India. It also has massive contracts with Indian companies to carry out X rays, diagnostic reporting, communication processes ... Lab work will also be transferred to India ... Doctors also send their recordings from their consultations, and overnight they are transcribed, printed and filed *(Respondent #6)

### Prospects

Over half of the respondents commented on the prospects for telemedicine trade. In general, most were optimistic about the prospects for telemedicine, either at the national or the international level. The UK stakeholders were particularly optimistic.

*There is no reason why information on some areas of treatment (X-ray, glucose levels) can't be transmitted to India ... There is real potential not to be bound by national borders *(Respondent #8)

However, the respondents thought that this will take time.

*The technology is here to stay. However, it will be evolutionary rather than revolutionary ... telemedicine will start in-country first, before going across borders *(Respondent #7)

The response from the Indian stakeholders was mixed. Whilst some believed there was great potential for telemedicine trade between the UK and India, others were of the opinion that the UK would not be their prime market, at least not in the near future.

*The size of the market is big for telemedicine ... The UK is not that big a potential market ... There is some potential for NHS outsourcing of radiology services but it will be quite a while before this happens and certainly not in the next five years or so *(Respondent #14)

*The best prospects are with countries in Africa and in other poor countries where there aren't sufficient number of trained people ... In the UK, the chances of doing telemedicine with them is not good *(Respondent #17)

Both sets of stakeholders agreed that the UK prefers the services to be delivered from the UK, even if this is done by a foreign provider. This is again contradictory to the notion that the UK is outsourcing some of its diagnostic services to India.

*The UK welcomes bids from providers from all countries that can deliver contracts, but need to provide services from the UK, to ensure quality and contract terms *(Respondent #1)

### Sectors

When asked about which segments of telemedicine have most potential for international trade, most respondents identified diagnostic services, especially radiology and pathology.

*Telemedicine can be used for treatment as well as for diagnostics ... The UK is not ready to use telemedicine for direct treatment for now *(Respondent #1)

*The segments of focus in India are tele-radiology and tele-pathology *(Respondent #10)

The UK stakeholders made a further distinction between telemedicine and tele-care, with the latter being favoured.

*In the UK in the last 5 years, the main opportunities have shifted from telemedicine to tele-care (supporting people in the community, monitoring them from the home) ... Tele-care is the major drive now ... This is part of the wider public health agenda, to support individuals in their homes. There is a policy agenda now of self-care and chronic disease management. In commercial terms, there has been recent growth in technologies that would facilitate these services ... internationally ... this has not been addressed in any major way *(Respondent #3)

*It *[tele-care] *can still be of use in the UK, as it would allow people to be monitored from home, rather than in hospital. They could talk daily to nurses on screens. There are plans to do this in the NHS, to use remote monitoring for chronic conditions, without the patient having to go in to hospital *(Respondent #6)

### Advantages

The stakeholders identified several advantages to importing health services via telemedicine, both for the UK and for India. In terms of the UK, participants identified saving time and money as the key advantages of telemedicine

*It is part of driving down cost and improving efficiency *(Respondent #6)

Whereas in India, asides from an increase in revenues, the importance of telemedicine trade in improving the national health system and the country image were highlighted as key advantages

*We could use telemedicine exports as a way to innovate and to catalyze and redesign our health system *(Respondent #10)

*The main benefits would be revenues, positive international exposure, opportunities to give back to the community in terms of training and corporate social responsibility work *(Respondent #15)

### Regulation, quality and litigation

One of the main concerns expressed by the respondents was whether the services offered by telemedicine would be of the same standard as those provided by the UK National Health Service.

*The main problem would be how to maintain standards ... in diagnostics, would there be a discrepancy in the result if someone had the test done in India or in the UK? The service could be potentially opening to a dual standard *(Respondent #5)

This was linked to problems and difficulties that would arise when trying to regulate health service providers that were based in a different country. Indian stakeholders recognised this as a barrier, and understood the need of being accredited. However, they showed discontent at the length and cost of the process. Additionally, there are concerns about litigation, and who would be responsible should there be any malpractice.

*In terms of treatment the EU law is unclear on responsibilities if something goes wrong *(Respondent #1)

*Where would any malpractice be tried, in the UK Court of Law or in the Indian Consumer Forum? *(Respondent #17)

### Other barriers

When asked about the potential barriers that may hinder an India-UK bi-lateral relationship in telemedicine, many stakeholders agreed that data safety would be the biggest worry.

*India has been deemed "data unsafe" by the EU, and in that respect the UK cannot make any contracts with them *(Respondent #1)

*We need to have a data protection law. The IT *[Information Technology] *act is not enough *(Respondent #10)

However, not everyone agreed about the importance of data safety, with some stakeholders thinking it was more of an excuse than a real issue.

*Data security is a made up issue. If it was a real issue the UK (or EU) would give India a list of things that have to improve for telemedicine trade to take place and India would oblige *(Respondent #9)

*There is a large volume of reporting that is done by South Africa for the UK and so why data protection is an issue for India is not clear *(Respondent #15)

One UK stakeholder did not even believe the EU had placed any restrictions on data safety:

*There are no barriers from the EU *(Respondent #6)

There was also some discussion on how this barrier could be overcome:

*The Indian government has to take the case to Brussels, and the UK would not stand in the way of that *(Respondent #1)

Another important concern for the UK stakeholders was public acceptance, with members of the Department of Health particularly worried a move to providing some services through telemedicine would prove unpopular with the British population.

*People wouldn't accept it; they would accuse the government of putting them at risk in order in order to cut costs *(Respondent #1)

*How it looks and feels to the end user is also very important. Would it be acceptable? They would see it as a cost-cutting measure; it would be an admission of failure *(Respondent #2)

Furthermore, some of the Indian respondents were also worried about the impact exporting telemedicine services would have on the local, Indian population.

*There is concern that there could be an adverse impact for the local health system. Doing tele-pathology or tele-radiology could reduce the quality of such services for the domestic population by diverting resources from domestic services *(Respondent #17)

Both the UK and Indian stakeholders interviewed identified protectionism and pressure from unions and the General Medical Council (GMC) as an important barrier for this type of trade in health services

*This *[telemedicine] *is unlikely to happen on a big scale unless there is some change around protectionism ... people, politics, money and greed *(Respondent #7)

*Professional organisations would also pose problems. The unions would raise objections based on risk and clinical competency, whilst these may be valid, the main reason would be job protection *(Respondent #7)

*It is important for doctors to trust their colleagues, which would be harder if they do not meet face to face, and they don't get to know them, know their background *(Respondent #8)

Finally, there were some concerns from the UK stakeholders that telemedicine may not deliver as good a service as traditional care.

*Telemedicine raises questions about the alternative, traditional methods of care: is it supporting people? Is it preventing important elements of clinical care? Is it going to limit person-person contact? Is something lost if doctor can't see/feel the patient? *(Respondent #3)

### Policy

The participants were asked about what policy changes would be needed for a UK-India trade relationship in telemedicine to be formalised. There was some disagreement between the stakeholders on this. Some of the UK participants believed no changes would be needed,

*There wouldn't need to be a policy change per se. It would be up to individual trusts to look at more innovative ways of working, keeping safety in mind *(Respondent #4)

whilst others thought a fundamental change in the NHS and Department of Health needs to take place:

*Changes are needed; it is a hearts and minds, money and skills operation. But they need to have the policy and authority to make changes to introduce telemedicine in a meaningful way, at the moment it is small, uncoordinated and fragmented ... Technologies moves quickly and the NHS very slowly! There is no pull mechanism, only push from industry *(Respondent #6)

From the Indian side, the healthcare providers that took part in the study felt that the government should be more involved in facilitating telemedicine exports, by improving India's image, working with private sector and facilitating trade.

*There should be support of telemedicine with incentives such as subsidies *(Respondent #11)

*There need to be certain initiatives, like establishing infrastructure of a certain standard, speed of communication, its dependability, and security *(Respondent #12)

However, this was opposed by the government:

*The government will not look at telemedicine for exports. This needs to be used domestically *(Respondent #19)

Additionally, many of the stakeholders felt policy solutions were needed for some of the barriers mentioned, including regulation, data protection and malpractice. In addition, some participants highlighted that these barriers would be better addressed through a bi-lateral trade relationship.

*The documentation requirements are very onerous. NHS processes need to be streamlined *...

*The GMC registration process should be simplified and expedited ... The Indian providers will need to show higher quality of work *(Respondent #15)

*Data protection ... could be addressed through bilateral agreements *(Respondent #10)

*There are issues of compensation in case of misdiagnosis and misreporting, though these can be taken care of through the contract and its enforcement *(Respondent #17)

## Discussion

The views of the stakeholders on the prospects for a bi-lateral trade relationship between the UK and India are summarised in table 3. The results from the semi-structured interviews show that there are a number of issues where stakeholders in both countries agree. These include good global prospects for trade in telemedicine services, the key sectors being diagnostic (mainly tele-radiology and tele-pathology), concerns regarding quality and litigation, the main advantages being saving time and money from the UK side, and generating revenues from the Indian side, and protectionism and lack of recognition being key barriers. These are also in agreement with the findings of the literature review previously carried out by the authors [11].

**Table 3:** Summary of stakeholder opinion on telemedicine trade between the UK and India

	UK	India
**Current activities**	Currently importing telemedicine services	Currently exporting telemedicine services

**Prospects for telemedicine trade**	Good	Good, but with the UK in the short-term

**Sectors**	Diagnostic services, tele-care	Mainly diagnostic services

**Advantages**	Lower costs of health care Increased efficiency	Higher revenues Improve health system Boost country image

**Barriers**	Quality Malpractice Public acceptance	Data safety Regulation Impact on local health care Protectionism

**Policy solutions**	Change in mentality	Government involvement Bi-lateral agreement

On the other hand, the participants disagreed on a number of issues. For instance, most of the UK and some of the Indian stakeholders believed that there were good prospects for a bi-lateral relationship in telemedicine services between the UK and India, whereas many of the Indian stakeholders were less optimistic. One important issue where there was disagreement was on the current activities of telemedicine cross-border trade taking place between the UK and India. Whilst some of the UK interviewees and most of the Indian participants believed there was no such trade taking place, other UK stakeholders argued that major contracts had been agreed by the NHS with Indian firms. This is an important issue to resolve as if this type of trade is indeed taking place, data can be collected on its viability and impact on both countries. It would also mean that these agreements have taken place despite the barriers mentioned in this study, and how this can take place should also be examined in more detail.. Other areas where there was disagreement include the importance of tele-care, which the Indian stakeholders were not aware of, whether data safety was a real barrier and what policy measures would be needed for a bi-lateral telemedicine trade relationship between the UK and India to be agreed.

It is interesting to see that some of the stakeholders called for a bi-lateral relationship to address some of the concerns and barriers involved with telemedicine trade. This is in agreement with the results of the literature review carried out by the authors [11], which concluded that in a bi-lateral relationship between the UK and India, both countries would sign a contract which would pre-establish the data safety procedures to be followed, the qualifications needed to provide services to UK patients, protocols in the event of malpractice, as well as mechanisms to ensure the local population in India and their health system benefit from the revenues such arrangement would bring. This would not be possible under a GATS-based multi-lateral trade scenario, where all countries can trade with each other and are not able to pre-establish this sort of pre-conditions.

This research is subject to several biases. First, although a common interview tool was used, the interviews were carried out by two different researchers, one based in India and one in the UK. This could have lead to questions being asked with different emphasis and answers recorded differently. Additionally, the sample size is small, and the opinion of the stakeholders that refused to take part, or any that may have not been identified, is therefore not included.

Nonetheless, some important policy recommendations can be made from the findings of this research. The main barrier reported was data safety and EU restrictions on dealing with India. It is therefore essential for the Indian government and companies planning on providing telemedicine services to the UK to ensure they are complying with the regulations and that other restrictions on this type of trade are lifted. Additionally, India should establish national data protection legislation and the UK (or the EU) should set up a system of accreditation for telemedicine providers. Other barriers include concerns regarding quality, regulation and litigation. Here, the UK should streamline and ease the processes required to be registered with the GMC, and India should aim for accreditation and quality assurance. In terms of sectors, it appears that telemedicine will mainly succeed for diagnostic services. Additionally, the UK stakeholders identified tele-care as a key sector, which the Indian health service providers should capitalise in. Further, if both countries were to enter into a bi-lateral agreement on trade in health services, they would need to outline litigation procedures to be followed should a case of malpractice arise, ensure the revenues generated are spent on India's health system, so the local population benefits, and choose the sectors where public resistance will be minimal (i.e. those where there is no interaction with the patient, such as pathology). Finally, telemedicine should also be raised in the ongoing EU-India trade negotiations, which cover trade in services between the EU and India.

## Competing interests

The authors declare that they have no competing interests.

## Authors' contributions

MMA contributed to the development of the survey instrument, carried out some of the interviews and drafted the initial paper and agreed on final version

RC conceptualised the work, contributed to the development of the survey instrument, carried out some of the interviews and reviewed, commented and agreed on final version

RS conceptualised the work, contributed to the development of the survey instrument, reviewed, commented and agreed on final version

## Supplementary Material

Additional file 1 Discussion guide used to conduct the interviews Click here for file
