# Supplementary material for: How is Telemedicine perceived? A qualitative study of perspectives from the UK and India
Source: Global Health. 2011 May 20;7:17. doi: 10.1186/1744-8603-7-17 (PMC3117690; doi:10.1186/1744-8603-7-17)
Supplement: Additional file 1 — Discussion guide used to conduct the interviews [file 1744-8603-7-17-S1.DOC]

**Additional file 1**

**Discussion Guide for Telemedicine Importers**

**Research Project on**

**Prospects for Telemedicine and Medical Tourism between India and the UK**

**I. Background information**

Name: ___________________________ Designation: _________________________

Name of organization: ______________________________________________________________

Location: _________________________________

Date and time: _________________________________

Contact address ___________________________________________________________

___________________________________________________________

Contact phone number/s: _________________________________

Email contact: _________________________________

Brief profile of organization: __________________________________________________________

__________________________________________________________________________________

Size: ___________________________

Turnover (optional): __________________________

**II. General Perspectives**

1. What are your views on the potential for telemedicine trade?

______________________________________________________________________________

______________________________________________________________________________

______________________________________________________________________________

______________________________________________________________________________

1. How promising do you think the prospects are for telemedicine trade at a global level?

Very high ____ Moderate ________ Low ________

Why?

_______________________________________________________________________________

______________________________________________________________________________

1. Which are the main regions and countries where you see prospects for such trade globally? List up to 5 in each case.

| Top 5 regions/  countries | Exporting regions | Exporting countries | Importing regions | Importing countries |
| --- | --- | --- | --- | --- |
| 1 |  |  |  |  |
| 2 |  |  |  |  |
| 3 |  |  |  |  |
| 4 |  |  |  |  |
| 5 |  |  |  |  |

1. Which are the main segments where you see prospects globally? (E.g., teleradiology) List up to 10.

______________________________________________________________________________

______________________________________________________________________________

1. If your list of importing countries in Q.3 above included the UK, how promising do you find the prospects for telemedicine imports to the UK?

Very high ____ Moderate ________ Low ________

Why?

_______________________________________________________________________________

_____________________________________________________________________________

1. In your view, what are the main sectors where these prospects lie for the UK? List up to 10

______________________________________________________________________________

______________________________________________________________________________

1. Which are the main markets to which the UK could outsource telemedicine services? List up to 10.

______________________________________________________________________________

______________________________________________________________________________

1. In your view, is the demand for telemedicine more likely to come from the private or from the public sector?

______________________________________________________________________________

______________________________________________________________________________

1. Are you aware of any barriers to telemedicine?

______________________________________________________________________________

______________________________________________________________________________

1. Are you aware of the discussions on trade in health services under the General Agreement on Trade in Services in the World Trade Organisation? Yes _______ No _______
2. Are you aware of the position of the UK government and of the NHS on telemedicine trade?

Yes ___ No ___

1. If yes, do you agree with the position? Yes fully _________ Yes to some extent _________ No _______

Why?

_______________________________________________________________________________

____________________________________________________________________________

1. Are there any disadvantages to telemedicine? Yes _______ No _______

If yes, which ones? ____________________________________________________________________

_________________________________________________________________________

1. What is the evidence base for the views expressed above, both generally and with specific regard to India and the UK?

Yes No

Secondary sources (newspaper and magazine reports, articles) _____ ______

Interactions with government organizations _____ ______

Interactions with industry players _____ ______

Conferences/seminars/meetings _____ ______

Operations and activities of your current/earlier organization/s _____ ______

Other (please state) __________________________________________________________

1. Are there any specific data sources you would recommend for information on telemedicine?

__________________________________________________________________________________

**III. Activities and Operations**

1. Does your organization purchase telemedicine services from overseas institutions?

Yes ____ No ____

2. If you answered No, please skip to Q. 9 below. If yes, please provide the following information.

|  | Numbers or shares | 2006 | 2007 | 2008 |
| --- | --- | --- | --- | --- |
| 1 | Total volume of services |  |  |  |
| 2 | Total no. of services outsourced[[1]](#footnote-2) |  |  |  |
| 3 | Proportion of services outsourced (%) |  |  |  |
| 4 | Savings from outsourcing telemedicine services[[2]](#footnote-3) |  |  |  |
| 5 | Share of total revenues accounted for by savings from outsourcing telemedicine savings |  |  |  |

1. List the top 5 countries from which you imported telemedicine services in each of the past three years and the associated numbers.

|  | 2006 | | 2007 | | 2008 | |
| --- | --- | --- | --- | --- | --- | --- |
|  | Country | No. of services[[3]](#footnote-4) | Country | No. of services^3^ | Country | No. of services^3^ |
| 1 |  |  |  |  |  |  |
| 2 |  |  |  |  |  |  |
| 3 |  |  |  |  |  |  |
| 4 |  |  |  |  |  |  |
| 5 |  |  |  |  |  |  |

1. What are the main segments in which you import telemedicine services? Tick all that are applicable below.

Telepathology ________

Teleradiology ________

Teledermatology ________

Telediagnostics ________

Telepsychiatry ________

Teleneurology ________

Teleeducation ________

Other ________

If you ticked “Other”, please list up to 5 other treatments ______________________________________________________________________________

1. If you have not listed India among your top 5 source countries for telemedicine imports, please answer the following:
2. Do you import from India? Yes ____ No ____

If no, skip to (c) below

1. If yes, how many services did you import from India (number or approximate share of all imports)? Also, list the top 3 treatments imported.

Numbers/shares: 2006 _______________ 2007 _________________ 2008 ____________

Treatments: __________________________, _______________________, _____________________

1. If no, do you still see India as a potential market for telemedicine imports?

High potential _________ Moderate potential ____________ No __________

Why?

___________________________________________________________________________

___________________________________________________________________________

1. What are the main challenges you have faced in outsourcing telemedicine services? List up to 5 challenges.

(a) _____________________________________________________________________________

(b) _____________________________________________________________________________

(c) **_____________________________________________________________________________**

(d) **_____________________________________________________________________________**

(e) **_____________________________________________________________________________**

1. Are there any challenges specific to outsourcing services to India? Yes ____ No _____

If you answered yes, list up to three such specific challenges.

1. ________________________________________________
2. _________________________________________________
3. _________________________________________________
4. What are the main benefits your organization has derived from telemedicine imports? List up to 3 such benefits.

____________________________ , _________________________________ , _________________________

1. If your organization does not import telemedicine services at this time, do you have plans to do so? Yes ____ No _____

Why?

___________________________________________________________________________

___________________________________________________________________________

**IV. Policy Issues**

1. How can telemedicine exports to the UK be increased? List a few specific steps that need to be taken by the governments on both sides.

In the UK In India

_______________________________________ ____________________________________

_______________________________________ ____________________________________

_______________________________________ ____________________________________

1. List up to 3 specific steps the industry could take in the UK to increase telemedicine services exports from India.

______________________________________________

______________________________________________

______________________________________________

1. List up to 3 specific steps your organization could take/is planning to take to increase telemedicine imports from India.

____________________________________________________

____________________________________________________

____________________________________________________

1. How optimistic are you about enhancing this bilateral relationship in telemedicine trade?

Very optimistic ______________ Moderately optimistic ______________ Not at all optimistic ______

Why?

_______________________________________________________________________________

____________________________________________________________________________

1. If you are moderately optimistic, in what kind of timeframe do you expect improved prospects?

____________________

1. Do you think partnerships and tie-ups or pilot arrangements could enhance telemedicine exports from India to the UK?

Yes ___________ No ___________

1. Do you think the India-EU Trade and Investment Agreement that is currently under negotiation could enhance prospects for telemedicine trade between the two countries?

Yes ____________ No ____________

List up to 3 specific issues that would need to be addressed under this agreement to enhance prospects for telemedicine exports from India to the UK.

_____________________________________________________________

_____________________________________________________________

_____________________________________________________________

1. Please list any other issues you would like to highlight and which have not been raised above?

______________________________________________________________________________

**Thank you**

1. If you are unable to provide absolute numbers of foreign cases, provide estimated shares in line 3. [↑](#footnote-ref-2)
2. If you are unable to provide absolute revenues from telemedicine exports, provide estimated shares in line 5. [↑](#footnote-ref-3)
3. If you are unable to provide absolute numbers, provide the share of foreign patients accounted for by each country. [↑](#footnote-ref-4)
